# Genome- and Proteome-Wide Analysis of Lysine Acetylation in *Vibrio vulnificus* Vv180806 Reveals Its Regulatory Roles in Virulence and Antibiotic Resistance

**DOI:** 10.3389/fmicb.2020.591287

**Published:** 2020-11-05

**Authors:** Rui Pang, Ying Li, Kang Liao, Penghao Guo, Yanping Li, Xiaojuan Yang, Shuhong Zhang, Tao Lei, Juan Wang, Moutong Chen, Shi Wu, Liang Xue, Qingping Wu

**Affiliations:** ^1^Guangdong Provincial Key Laboratory of Microbial Safety and Health, State Key Laboratory of Applied Microbiology Southern China, Guangdong Institute of Microbiology, Guangdong Academy of Sciences, Guangzhou, China; ^2^Department of Laboratory Medicine, The First Affiliated Hospital of Sun Yat-sen University, Guangzhou, China; ^3^College of Food Science, South China Agricultural University, Guangzhou, China

**Keywords:** *Vibrio vulnificus*, whole genome sequencing, acetylome, virulence, antibiotic resistance

## Abstract

Infection with *Vibrio vulnificus* is notorious for its atypical clinical manifestations and irreversible disease progression. Lysine acetylation is a conserved post-translational modification (PTM) that plays a critical regulatory role in diverse cellular processes. However, little is known about the role of lysine acetylation on the pathogenesis of *V. vulnificus*. Here, we report the complete genome sequence and a global profile for protein lysine acetylation of *V. vulnificus* Vv180806, a highly cefoxitin resistant strain isolated from a mortality case. The assembled genome comprised two circular chromosomes and one circular plasmid; it contained 4,770 protein-coding genes and 153 RNA genes. Phylogenetic analysis revealed genetic homology of this strain with other *V. vulnificus* strains from food sources. Of all the proteins in this strain, 1,924 (40.34%) were identified to be acetylated at 6,626 sites. The acetylated proteins were enriched in metabolic processes, binding functions, cytoplasm, and multiple central metabolic pathways. Moreover, the acetylation was found in most identified virulence factors of this strain, suggesting its potentially important role in bacterial virulence. Our work provides insights into the genomic and acetylomic features responsible for the virulence and antibiotic resistance of *V. vulnificus*, which will facilitate future investigations on the pathogenesis of this bacterium.

## Introduction

*Vibrio vulnificus* is an opportunistic gram-negative pathogen that is widely distributed throughout marine and brackish environments (Gulig et al., 2005). This bacterium can cause severe gastroenteritis from raw seafood consumption, as well as wound infections and necrotizing fasciitis, with mortality rates for sepsis and wound infection up to 50 and 17%, respectively (Horseman and Surani, 2011). Most deaths occur in patients with pre-existing conditions, such as those with compromised immune system or elevated serum iron levels (primarily in alcohol-associated liver cirrhosis) (Gulig et al., 2005; Oliver, 2005). Despite the rarity of infections, *V. vulnificus* is responsible for up to 94% of the deaths caused by non-cholera *Vibrios* (Jones and Oliver, 2009). Moreover, the incidence of *V*. *vulnificus* infection has risen worldwide, due to the ongoing increase in sea surface temperatures associated with global warming (Baker-Austin et al., 2013). Significantly, a large range of genomic diversity was observed for pathogenic *V*. *vulnificus* strains (López-Pérez et al., 2019). Though multiple virulence factors (VFs) (e.g., hemolysin, capsular polysaccharide [], etc.) have been identified independently, the regulation of these VFs in the pathogenicity of *V*. *vulnificus* is still largely unknown (Horseman and Surani, 2011; Phillips and Satchell, 2017).

Post-translational modifications (PTMs) are critical regulatory mechanisms for gene expression that occur during or after protein biosynthesis in diverse cellular processes (Walsh et al., 2005; Cain et al., 2014). Among various PTMs, lysine acetylation is one of the most common PTMs in all kingdoms of lives (Choudhary et al., 2009; Wu et al., 2011; Henriksen et al., 2012). Lysine acetylation, including histone acetylation and non-nuclear protein acetylation, is a dynamic and reversible process. Recent developments in high resolution mass spectrometry and the advent of specific purification methods have allowed large-scale analyses of protein acetylation, and have provided valuable insights into protein regulation. To date, the proteome-wide lysine acetylation profiles have been analyzed for a number of eukaryotes and prokaryotes, including important bacterial pathogens such as *Escherichia coli* (Zhang et al., 2009, 2013; Schilling et al., 2019), *Staphylococcus aureus* (Zhang et al., 2014), *Vibrio parahaemolyticus* (Pan et al., 2014), *Mycobacterium tuberculosis* (Xie et al., 2016b), *Vibrio cholerae* (Jers et al., 2017), *Pseudomonas aeruginosa* (Gaviard et al., 2018), *Salmonella enterica* (Li et al., 2018), and *Streptococcus pneumoniae* (Liu et al., 2018). These studies implied that protein lysine acetylation might play important roles in bacterial pathogenesis and drug resistance (Ren et al., 2017; Li et al., 2018). However, to date, protein acetylation in *V*. *vulnificus* is poorly studied.

In the present study, we report the complete genome sequence and the lysine acetylome of *V*. *vulnificus* strain Vv180806, which was isolated from the blood culture of a patient who died due to a necrotizing fasciitis infection in Guangzhou (Guangdong province of China). The case might be a representative food-borne infection because that no traceable traumatic history was found in the patient. In addition, this strain showed high level of resistance to the beta-lactam antibiotic cefoxitin. Antibiotic resistance of *V*. *vulnificus* is not as well studied as other bacterial pathogens, though its antibiotic resistance has been frequently reported in recent years (Elmahdi et al., 2016). Hence, we characterized the genomic features of this strain with potential risk of food-borne infection to reveal the molecular mechanisms underlying its antibiotic resistance and virulence. Furthermore, proteome-wide analysis was performed to determine the potential role of lysine acetylation in the virulence and antibiotic resistance of *V*. *vulnificus* Vv180806. To the best of our knowledge, this is the first genomic map of lysine acetylation for *V*. *vulnificus*, which will facilitate further investigation concerning the development of antibiotic resistance and the pathogenicity of this harmful pathogen.

## Materials and Methods

### Bacterial Strain and the Assessment of Antibiotic Resistances

*V*. *vulnificus* Vv180806 was isolated from the blood culture of a patient with necrotizing fasciitis in Guangzhou, Guangdong province of China, on 6 August 2018. The antibiotic resistance profiles were assessed through minimal inhibitory concentration (MIC) assays (Wiegand et al., 2008). Briefly, a final suspension of ∼5 × 10^5^ cells/ml in broth supplemented with 2% NaCl and 1 mM CaCl_2_-H_2_O were distributed in triplicate throughout a 96-well microtiter plate. *Escherichia coli* ATCC25922 was used as the susceptible-control reference bacterial strain for MIC assays. Cells were challenged with 0.5–1,024 g/ml antibiotics. MICs were determined by detection of cell pellet formation in the bottom of the wells of the 96-well plate; the results were corroborated colorimetrically with alamarBlue vital dye (Invitrogen, United States), following the manufacturer’s protocol.

### Extraction of Genomic DNA and Whole Genome Sequencing

The genomic DNA of isolate Vv180806 was extracted using a genomic DNA extraction kit (Magen Biotech, Guangzhou, China) according to the manufacturer’s instructions. The quality and integrity of the genomic DNA was assessed using 0.8% agarose gel electrophoresis, where a single band of approximately 50 kb was expected. Meanwhile, DNA concentration and purity were measured using a NanoDrop 2,000 spectrophotometer (Thermo Fisher Scientific, United States) and Qubit 3.0 fluorometer (Thermo Fisher Scientific, United States).

Two different genomic DNA libraries were constructed according to the manufacturers’ instructions of the Illumina Hiseq platform and PacBio Sequel platform. For Illumina Hiseq sequencing, genomic DNA was first fragmented into 500 bp using the Covaris M220 sonicator (Covaris, Woburn, United States). The Ultra^TM^ DNA Library Prep Kit for Illumina (NEB, United States) was then employed for the generation of genomic DNA libraries, with the end-reparation, adaptor ligation with Illumina adapters, size selection with beads, and enrichment steps followed according to the manufacturer’s instructions. The libraries were assessed using an Agilent Bioanalyzer 2,100 (Agilent Technologies, Palo Alto, CA, United States) and a Qubit 3.0 fluorometer. The Illumina Hiseq platform sequencing with Paired-end (PE) 150 bp was performed by GENEWIZ Ltd. (Suzhou, China). For PacBio Sequel sequencing, DNA samples were fragmented into 10 kb using a Covaris g-TUBE shearing device and then purified with AMPure XP Beads (Beckman Coulter, United States). The fragmented genomic DNA was used to construct the library with the PacBio SMRTbell library preparation kit according to manufacturer’s instructions. The SMRTbell libraries were then selected using BluePippin (Sage Science, Beverly, MA, United States) and were sequenced using the PacBio Sequel platform by GENEWIZ Ltd.

### Genome Assembly and Annotation

Low quality reads from Illumina sequencing were filtered out using Cutadapt software (Martin, 2011). For PacBio long reads, the assembly was performed using the hierarchical genome-assembly process 4 (HGAP4) pipeline (Pacific Biosciences, SMRT Link 5.0). The filtered Illumina reads were aligned to the *de novo* assembled contigs using the Burrows-Wheeler Alignment software (Li and Durbin, 2009). The alignment results were sorted by Picard^1^, followed by base quality recalibration with Pilon (Walker et al., 2014). The final contigs were circularized by Circlator version 1.5.5 (Hunt et al., 2015), and the completeness of the genomics data was assessed by BUSCO (Simao et al., 2015). The whole genome sequence of *V*. *vulnificus* Vv180806 has been deposited in GenBank under accession numbers CP044206–CP044208. All raw reads of the *V*. *vulnificus* Vv180806 genome have been deposited in the NCBI Sequence Read Archive under accession number SRR10161471.

Gene annotation of the whole genome sequence was conducted through the Prokka v1.11 (Seemann, 2014). The protein-coding genes were then annotated in other public databases including NR (Non-redundant), KEGG (Kyoto Encyclopedia of Genes and Genomes), COG (Clusters of Orthologous Groups), GO (Gene Ontology), eggNOG (Huerta-Cepas et al., 2019), VFDB (Chen et al., 2016), CARD (Jia et al., 2017), and Resfinder (Zankari et al., 2012). The potential virulence factors (VFs) were further identified according to previous references (Gulig et al., 2005; Jones and Oliver, 2009; Horseman and Surani, 2011).

### Comparative Genomics Analysis

All genome sequences of *V*. *vulnificus* were downloaded from the National Centre for Biotechnology Information (NCBI) databases (up to 1 March 2019). The genome sequences were re-annotated with Prokka v1.11 (Seemann, 2014), and the pan-genome analysis was conducted based on the output of Prokka by using Roary v3.11.2 with a BLASTP identity cutoff of 90% (Page et al., 2015). Whole genome average nucleotide identity (ANI) between pairwise *V*. *vulnificus* strains were calculated with Pyani software available at https://github.com/widdowquinn/pyani. The core genome of these strains was produced by Harvest software v1.1.2 (Treangen et al., 2014) using the CMCP6 genome as a reference. After the core-genome alignment was generated, Gubbins was used to conduct recombination analysis and remove the putative recombined regions (Croucher et al., 2015). Single nucleotide polymorphisms (SNPs) were then extracted from the recombination-free core-genome alignment using the script available at https://github.com/sanger-pathogens/snp-sites. The maximum likelihood (ML) phylogenetic tree was constructed on the concatenated core SNPs using RAxML v8.2.10 in the GTRGAMMA model (1,000 bootstrap) (Stamatakis, 2014) and was visualized using iTOL (Letunic and Bork, 2016).

### Bacterial Growth Condition and Protein Exaction

*V*. *vulnificus* Vv180806 was inoculated in fresh lysogeny broth medium, 3% alkaline peptone water medium, and artificial seawater, respectively. After an overnight incubation at 37°C, the cells from different media were harvested at an OD600 of 0.6 by centrifugation at 10,000 g for 10 min at 4°C and combined into two biological replicates for protein exaction.

The harvested cells were sonicated three times on ice using a high intensity ultrasonic processor (Scientz) in lysis buffer (8 M urea, 1% Protease Inhibitor Cocktail). The remaining debris was removed by centrifugation at 12,000 g at 4°C for 10 min. Finally, the supernatant was collected and the protein concentration was determined with BCA protein assay kit (Beyotime, Shanghai, China) according to the manufacturer’s instructions.

### Protein Digestion and Affinity Enrichment of Acetylated Peptides

For digestion, the protein extracts were reduced with 5 mM dithiothreitol for 30 min at 56°C and alkylated with 11 mM iodoacetamide for 15 min at room temperature in darkness. The protein sample was then diluted by adding 100 mM triethylammonium bicarbonate to urea concentration less than 2M. Finally, trypsin was added at 1:50 trypsin-to-protein mass ratio for the first digestion overnight and 1:100 trypsin-to-protein mass ratio for a second 4 h-digestion. The tryptic peptides were fractionated into fractions by high pH reverse-phase High Performance Liquid Chromatography (HPLC) using Thermo Betasil C18 column (5 μm particles, 10 mm ID, 250 mm length). Briefly, peptides were first separated with a gradient of 8–32% acetonitrile (pH 9.0) over 60 min into 60 fractions. Then, the peptides were combined into 4 fractions and dried by vacuum centrifuging.

To enrich acetylated peptides, tryptic peptides dissolved in NETN buffer (100 mM NaCl, 1 mM EDTA, 50 mM Tris-HCl, 0.5% NP-40, pH 8.0) were incubated with pre-washed pan-anti-acetyl lysine antibody beads (PTM Bio, Hangzhou, China) at 4°C overnight with gentle shaking. Then the beads were washed four times with NETN buffer and twice with H_2_O. The bound peptides were eluted from the beads with 0.1% trifluoroacetic acid. Finally, the eluted fractions were combined and vacuum-dried. For liquid chromatography-tandem mass spectrometry (LC-MS/MS) analysis, the resulting peptides were desalted with C18 ZipTips (Millipore) according to the manufacturer’s instructions.

### Liquid Chromatography-Tandem Mass Spectrometry Analysis

The tryptic peptides were dissolved in 0.1% formic acid (solvent A), directly loaded onto a home-made reversed-phase analytical column (15 cm length, 75 μm i.d.). The gradient was comprised of an increase from 6 to 23% solvent B (0.1% formic acid in 98% acetonitrile) over 26 min, 23–35% in 8 min and climbing to 80% in 3 min then holding at 80% for the last 3 min, all at a constant flow rate of 400 nL/min on an EASY-nLC 1,000 Ultra Performance Liquid Chromatography (UPLC) system.

The peptides were subjected to Nano spray ion source followed by tandem mass spectrometry (MS/MS) in Q ExactiveTM Plus (Thermo Fisher Scientific) coupled online to the UPLC. The electrospray voltage applied was 2.0 kV. The m/z scan range was 350–1,800 for full scan, and intact peptides were detected in the Orbitrap at a resolution of 70,000. The m/z scan range for the secondary scan was set to 100 and the fragments were detected in the Orbitrap at a resolution of 17,500. A data-dependent procedure that alternated between one MS scan followed by 20 MS/MS scans with 15.0 s dynamic exclusion. Automatic gain control was set at 5E4. Fixed first mass was set as 100 m/z. The mass spectrometry proteomics data have been deposited to the ProteomeXchange Consortium via the PRIDE (Perez-Riverol et al., 2019) partner repository with the dataset identifier PXD020376.

### Database Search

The resulting MS/MS data were processed using Maxquant search engine (v.1.5.2.8). Tandem mass spectra were searched against the total protein sequences of *V*. *vulnificus* Vv180806 concatenated with reverse decoy database. Trypsin/P was specified as cleavage enzyme allowing up to four missing cleavages. The mass tolerance for precursor ions was set as 20 ppm in First search and 5 ppm in Main search, and the mass tolerance for fragment ions was set as 0.02 Da. Carbamidomethyl on Cys was specified as fixed modification, and lysine acetylation and oxidation on Met were specified as variable modifications. FDR was adjusted to < 0.01 and minimum score for modified peptides was set > 40. Only proteins that were detected to be acetylated in both replicates were selected for subsequent bioinformatics analysis.

### Bioinformatics Analysis of Acetylome Data

Motif-X was used for analyzing the models of protein sequences constituted with amino acids in specific positions (10 amino acids upstream and downstream of the acetylated modification site) (Chou and Schwartz, 2011). GO enrichment analysis was performed in Blast2GO with Fisher’s exact test of FDR < 0.05. KEGG pathway enrichment analysis was performed by a two-tailed Fisher’s exact test (adjusted *P* < 0.05). For protein-protein interaction network analysis of acetylated VFs and antibiotic resistance genes, interaction data (score > 0.4) from the STRING database^2^ was retrieved and visualized with Cytoscape (version 3.5.1) (Shannon et al., 2003).

## Results and Discussion

### General Features of the Strain Vv180806

The antibiotic resistance pattern of *V*. *vulnificus* Vv180806 is presented in Table 1. This isolate was susceptible to all tested antibiotics except cefoxitin. While cefoxitin resistance has been reported in zoonotic *V*. *vulnificus* (Haenen et al., 2014), its occurrence in clinical cases has rarely been reported. In addition, the gene responsible for cefoxitin resistance in *V*. *vulnificus* remains unknown.

**TABLE 1:** The antibiotic resistance profile of *V. vulnifcus* Vv180806.

Antibiotics	MIC (μg/ml)	Susceptibility
Amoxicillin/CA	≤ 2	S
Amikacin	4	S
Cefoperazone	≤ 8	S
Ceftriaxone	≤ 0.25	S
Cefepime	0.25	S
Cefoxitin	≤ 64	R
Imipenem	≤ 0.25	S
Levofloxacin	≤ 0.12	S
Cefuroxime-Sodium	4	S
Cefuroxime-Axetil	4	S
Trimethoprim	≤ 20	S
Ceftazidime	0.25	S
Tigecycline	≤ 0.5	S
Piperacillin	≤ 4	S

**S: Susceptible; R: Resistant.**

We used both Illumina and PacBio technologies to sequence the whole genome to explore the genomic features responsible for the high level of cefoxitin resistance and virulence of this isolate. The complete genome of *V*. *vulnificus* Vv180806 comprised two chromosomes, chr1 and chr2, with lengths of 3,400,045 and 1,890,883 bp, respectively, and had an average GC content of 46.62% (Supplementary Figure S1). This isolate also possessed a plasmid that had a length of 65,574 bp and an average GC content of 43.56%. Taken together, the complete genome was predicted to contain 4,770 protein-coding genes, 34 rRNAs, and 119 tRNAs.

**FIGURE 1:**
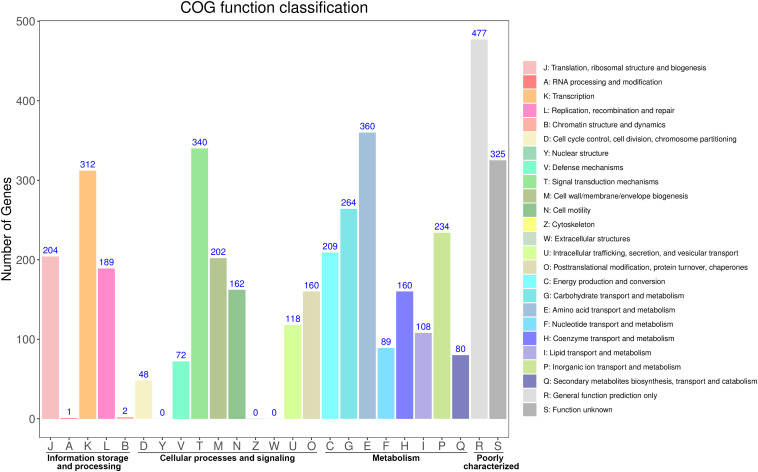
Functional classification of the protein coding genes in *V. vulnificus* Vv180806 (COG classification).

The distribution of these protein-coding genes into COGs functional categories is shown in Figure 1. Except for genes with unknown functions, most genes were found to be related with transcription, amino acid transport and metabolism, signal transduction mechanisms, carbohydrate transport and metabolism, cell wall/membrane/envelope biogenesis, and replication, recombination and repair. GO annotation revealed that most of these genes were enriched in the functional categories of catalytic activity, metabolic processes, and membrane maintenance (Supplementary Figure S2). The functional categories included multiple stress response proteins, suggesting that the strain Vv180806 has a strong adaptive capacity to environmental or host stresses.

### Identification of the Virulence and Antibiotic Resistance Genes

**FIGURE 2:**
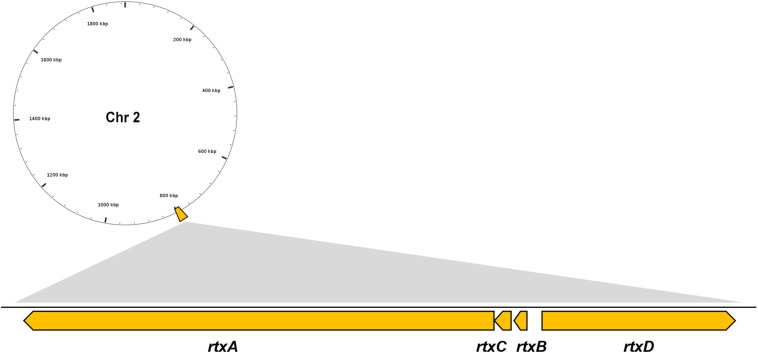
Genomic architecture of the RTX virulence factors in *V. vulnificus* Vv180806. *rtxA*, RTX toxins determinant A; *rtxB*, RTX toxin transporter B; *rtxC*, RTX toxin activating lysine-acyltransferase; *rtxD*, RTX toxin transporter D.

Multiple VFs are found in the genome of *V*. *vulnificus* Vv180806 according to the BLASTed search of VFDB and the previous reports (Supplementary Table S1). There is a gene cluster that accounts for the production of RTX toxin (Figure 2), which has been shown to play an important role in the pathogenesis of *V. vulnificus* infections (Liu and Crosa, 2012). Besides, two regulators (HlyU and RpoS), which were reported to regulate RtxA toxin (Liu and Crosa, 2012; Guo et al., 2018), are found in the Chr 1 of Vv180806. Additionally, the gene encoding hemolysin/cytolysin (VvhA) is also found in Chr 2 of Vv180806. This cytotoxin is associated with invasiveness and damage of infected tissues (Park et al., 1996). The existence of outer membrane protein U (OmpU) and immunogenic lipoprotein A (IlpA) indicated that the bacterial adhesion might also play an important role in the infection of this strain (Goo et al., 2006; Lee et al., 2010). Other regulatory factors like ferric uptake regulator (Fur), SmcR, and LuxS, might impact the pathogenesis through the regulation of quorum sensing of *V*. *vulnificus* (Pajuelo et al., 2016; Wen et al., 2016). Thus, the death of this infection case has strong relationship with a mixing effect of multiple virulence factors.

At least 4 antibiotic resistance-related genes, including *CRP*, *varG*, *tet(34)* (encoding an oxytetracycline resistance phosphoribosyltransferase) and *tet(35)* (encoding a tetracycline efflux pump), were identified in the genome of Vv180806 (Supplementary Table S2). CRP (cAMP-cAMP receptor protein) is a global regulator that not only regulates the expression of multidrug efflux pump but also impacts the expression of multiple VFs (Nishino et al., 2008; Zhan et al., 2008). Notably, VarG has been shown to have beta-lactamase activity against penicillin, carbapenems, and cephalosporins *in vitro* (Lin et al., 2017), which might account for the cefoxitin resistance of this bacterial strain. The presence of other resistant genes also implied the potential development of other antibiotic resistance phenotypes in this strain.

### Comparative Genomics Analysis

**FIGURE 3:**
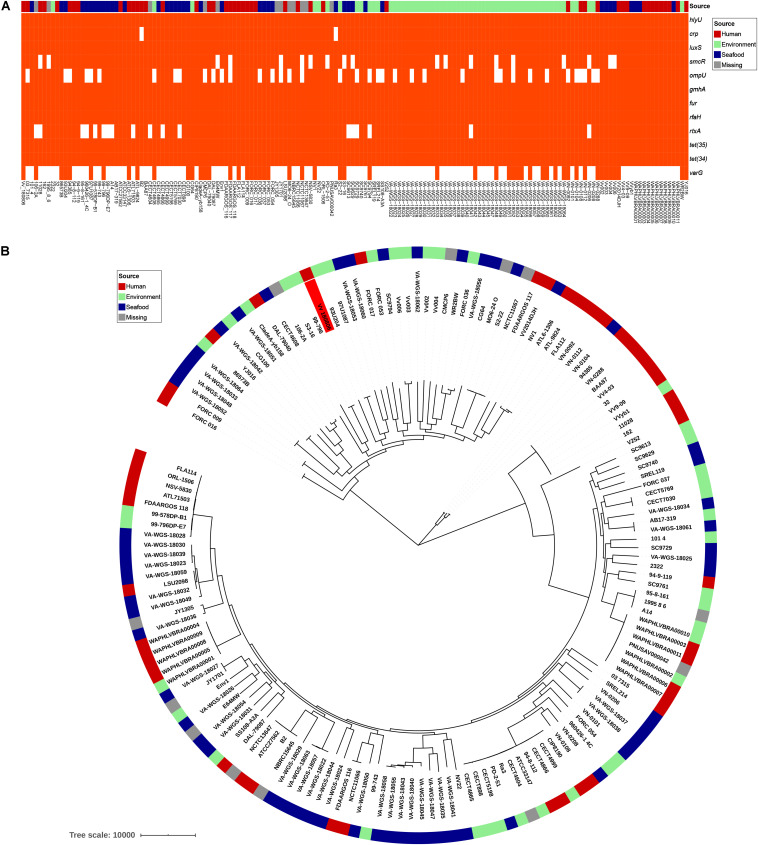
Comparative genomic analysis of *V. vulnificus* Vv180806 with other *V. vulnificus* strains. **(A)** Distribution of potential virulence genes and antibiotic resistant genes among *V. vulnificus* Vv180806 with other *V. vulnificus* strains. *hlyU*, the hemolysin gene transcription activator; *crp*, cAMP-activated global transcriptional regulator; *luxS*, S-ribosylhomocysteine lyase; *smcR*, regulator of quorum sensing gene; *ompU*, outer membrane protein; *gmhA*, phosphoheptose isomerase; *fur*, iron-ferric uptake regulator; *rfaH*, regulator of transcript elongation; *rtxA*, RTX toxins determinant A; *tet(35)*, tetracycline efflux pump; *tet(34)*, oxytetracycline resistance phosphoribosyltransferase; *varG*, ambler class B metallo-beta-lactamase. **(B)** Maximum-likelihood phylogeny of *V. vulnificus* Vv180806 with other *V. vulnificus* strains based on core genome single nucleotide polymorphisms. The red color in the clades indicate strain Vv180806, and the bar colors indicate the isolated sources of each strain.

*V*. *vulnificus* Vv180806 shared a total of 2,034 core genes (> 99% presence) and 729 soft-core genes (> 95 and ≤ 99% presence) with other *V. vulnificus* strains according to the pan-genome analysis (Supplementary Figure S3). The VFs *hlyU*, *luxS*, *gmhA*, *fur*, and *rfaH*, and the antibiotic resistant genes *tet*(34) and *tet*(35), were included in core genes, and *CRP* was attributed to soft-core gene (Figure 3A). *smcR*, *ompU*, and *rtxA* were present in 76–89% of the analyzed *V. vulnificus* strains, suggesting the prevalence of these VFs. The sequence of potential cefoxitin resistance-related gene *varG* was only present in 33% of the analyzed *V. vulnificus* strains, and most of the strains carrying this gene were isolated from environment and seafood. The cefoxitin resistance of *V*. *vulnificus* was previously reported in aquacultures (García-Aljaro et al., 2014; Haenen et al., 2014), and our finding reinforces the need for surveillance of antibiotic resistant *V*. *vulnificus* in seafood and aquatic environments. Besides, we identified 153 strain-specific genes in *V*. *vulnificus* Vv180806, the count of which were obviously higher than the average strain-specific gene number (73) of all analyzed *V. vulnificus* strains (Supplementary Table S3). The specific genes in strain Vv180806 were involved in functional categories of replication/recombination/repair, defense mechanism, cell wall/membrane/envelope biogenesis (Supplementary Figure S4). The high proportion of specific genes and stimulus-related functions of these genes might render a strong adaptive capacity of this strain.

The average ANI value between strain Vv180806 and other *V. vulnificus* strains was 96.18% (range from 95.04 to 98.39%) (Supplementary Figure S5). The most similar strain to Vv180806 was FORC_036 (identity 98.39%), a strain isolated from seafood. This was in accordance with the case report of this bacterial strain that indicated a potential food-borne infection. The phylogenetic tree of the strain Vv180806 and other *V. vulnificus* strains, constructed on the basis of the core genome alignment (Figure 3B), was consistent with the previous clustering of all *V. vulnificus* strains (López-Pérez et al., 2019); Vv180806 was grouped with one of the largest *V. vulnificus* clusters exhibiting a bloomer ecotype (typical of microbes that take advantage of sporadic organic matter availability in the environment for their growth). Notably, the genetic homology of Vv180806 and seafood source isolates further suggested the risk of food-borne infections. Infection with *V. vulnificus* due to the consumption of contaminated seafood has been previously reported (Phillips and Satchell, 2017). Our analysis reinforces the need for surveillance of this pathogen in consumptive seafood.

### Comprehensive Analysis of Lysine Acetylated Proteins in *V. vulnificus*

According to the annotation results of NR and eggNOG databases, a total of 51 acetyltransferases and 7 deacetylases were identified from the genome of *V*. *vulnificus* Vv180806 (Supplementary Table S4). The numbers of these two enzymes are higher than those previously identified in *V. cholerea* (Jers et al., 2017), indicating a remarkably important role of acetylated modification in *V*. *vulnificus*. Thus, a large-scale proteomic study was applied to identify the genome-wide lysine acetylation in *V*. *vulnificus* Vv180806. The high-resolution LC-MS/MS analysis yielded a total of 6,626 high-confidence acetylation sites on 1,924 proteins that occupied 40.34% of the total proteins in this strain (Supplementary Table S5). To our knowledge, this is the largest acetylated protein number that have been identified in bacteria (Christensen et al., 2019), and the ratio of acetylated proteins is higher than all previously identified ratios in other bacteria except for that in *Spiroplasma eriocheiris* (44.69%) (Meng et al., 2016). There may be three explanations for this observation. First, the potential opportunistic lifestyle of this *V. vulnificus* strain may drive it to utilize any possible source to fulfill its survival and growth (López-Pérez et al., 2019), thus this bacterium will tend to mobilize more functional proteins by acetylation. Second, the improvement of antibody specificity and mass spectrometry sensitivity, and the development of proteomic technologies, will certainly benefit to the identification of lysine acetylation in bacterial cells (Pan et al., 2014; Christensen et al., 2019). Third, the lysine acetylated proteins were searched against a completed genome sequences of the same bacterial strain, which might increase the accuracy of sequence searching. Therefore, the highest number of acetylated proteins identified in our present study is reasonable.

**FIGURE 4:**
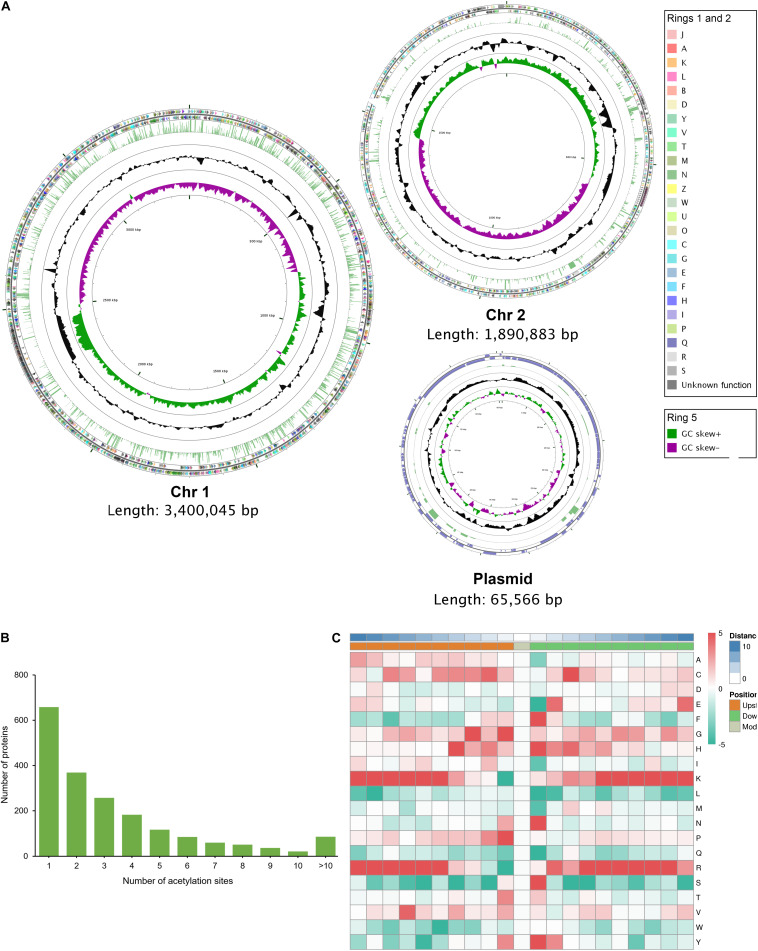
The genome-wide profile of lysine acetylation in *V. vulnificus* Vv180806. **(A)** Circular representation of the genome-wide profile of lysine acetylation. The outer two rings (ring 1 and ring 2) represent the annotated genes encoding proteins on the plus and minus strands, respectively, and different colors represent different COG categories for the corresponding genes. COG categories: [A] RNA processing and modification; [B] Chromatin structure and dynamics; [J] Translation, ribosomal structure and biogenesis; [K] Transcription; [L] Replication, recombination and repair; [D] Cell cycle control, cell division, chromosome partitioning; [O] Posttranslational modification, protein turnover, chaperones; [M] Cell wall/membrane/envelope biogenesis; [N] Cell motility; [P] Inorganic ion transport and metabolism; [T] Signal transduction mechanisms; [U] Intracellular trafficking, secretion, and vesicular transport; [V] Defense mechanisms; [W] Extracellular structures; [Y] Nuclear structure; [Z] Cytoskeleton; [C] Energy production and conversion; [G] Carbohydrate transport and metabolism; [E] Amino acid transport and metabolism; [F] Nucleotide transport and metabolism; [H] Coenzyme transport and metabolism; [I] Lipid transport and metabolism; [Q] Secondary metabolites biosynthesis, transport and catabolism; [R] General function prediction only; [S] Function unknown. Ring 3 indicates the lysine acetylated proteins and the height of laurel-green bar is determined according to the number of acetylation sites. Ring 4 (black circle) indicates the GC content (%), and ring 5 depicts the GC skew. **(B)** Distribution of acetylation sites in each acetylated protein. **(C)** The motif enrichment heatmap of upstream and downstream amino acids of all identified modification sites. Red indicates that this amino acid is enriched near the modification site, and green indicates that this amino acid is reduced near the modification site.

The distribution of acetylated proteins is disequilibrium across the genome of strain Vv180806. The proportion of protein acetylation in Chr1 is 44.72% (1431/3200), which is obviously higher than those in Chr2 (28.45%, 468/1645) and in plasmid (31.65%, 25/79) (Figure 4A). This phenomenon may be the result of that Chr1 harbors more functional proteins (68.94%, 2206/3200) than Chr2 (59.57%, 980/1645) and plasmid (15.19%, 12/79) according to the COG annotation (Figure 4A). In addition, Chr 1 encodes more house-keeping genes such as translation, ribosomal structure and biogenesis-related genes. Of the 1,924 acetylated proteins, 65.80% contain two or more acetylation sites, and 4.47% contain more than 10 acetylation sites (Figure 4B). This proportion is also slightly higher than that found in previous studies (Jers et al., 2017; Li et al., 2020; Pang et al., 2020), while further experiments are required to validate these acetylated proteins in the future. We further determined the frequencies of amino acids around the acetylated lysine site (Supplementary Table S6 and Supplementary Figure S6). Phenylalanine, histidine, asparagine, serine, and tyrosine are preferentially located at the + 1 position, and glycine and proline at the -1 position (Figure 4C). For all ten amino acids upstream and downstream of the modification site, lysine and arginine are the most overrepresented ones, especially in positions −10 to −5 and + 3 to + 10. This is in similar to the previous finding in *V. cholerae* (Jers et al., 2017), indicating the conversation of motif preference in *Vibrio* spp.

### Functional Annotation and Enrichment of Acetylated Proteins in *V. vulnificus*

**FIGURE 5:**
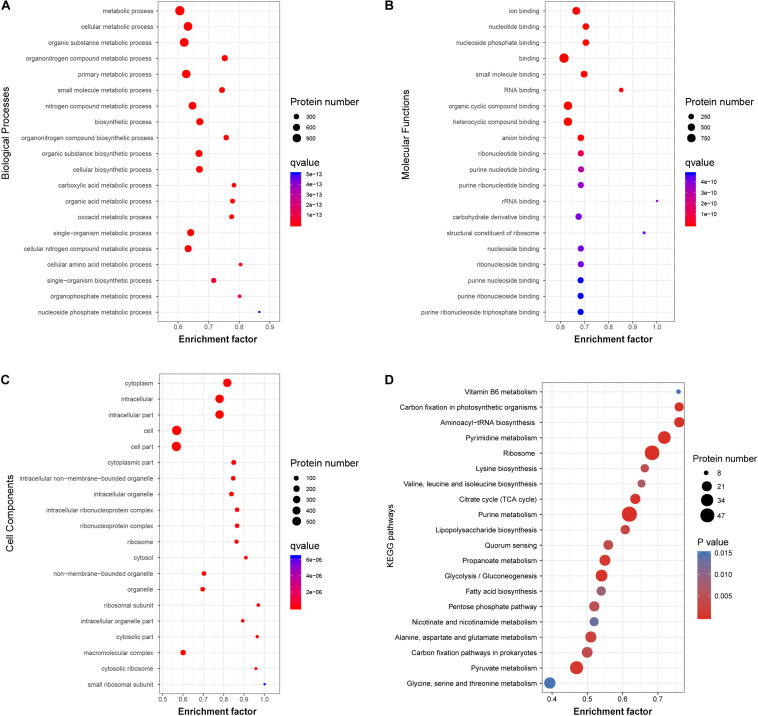
Functional classification of acetylated proteins in *V. vulnificus* Vv180806. **(A–C)** Biological Processes, Molecular Functions, and Cell Components enrichment analysis of the acetylated proteins. **(D)** The enrichment of the acetylated proteins in KEGG pathway. Enrichment Factor: The number of acetylated proteins divided by the number of total proteins in corresponding category.

To investigate the functional role of the acetylated modification in *V. vulnificus* Vv80806, the enrichment in GO functional categories and KEGG pathways of acetylated proteins were analyzed (Supplementary Tables S7–S10). Accordingly, the biological process (BP) categories of metabolic, especially of cellular, organic substance, and organonitrogen compound metabolic process, are significant enriched for these proteins (Figure 5A). In the molecular function (MF) categories, most of the acetylated proteins are related to the ion binding, nucleotide binding, nucleoside phosphate binding, small molecule binding, and RNA binding (Figure 5B). The significantly enriched cellular component (CC) categories contain cytoplasm, intracellular, cell, etc. (Figure 5C). These functional enrichments are largely consistent with previous findings which showed that most lysine acetylated proteins are categorized as involved in metabolic processes, binding functions and location of the cytoplasm (Pan et al., 2014; Jers et al., 2017; Liu et al., 2018).

The KEGG pathway enrichment analysis of acetylated proteins revealed the overrepresentation of central metabolic pathways, including citric acid (TCA) cycle, purine metabolism, and pyrimidine metabolism (Figure 5D). This observation is in accord with those in other organisms and reflects a regulatory role of acetylation in central metabolism (Xie et al., 2016a; Schilling et al., 2019). Notably, the enrichment of carbohydrate metabolism and energy metabolism pathways indicates that acetylated modification may play an important role in the carbon source utilization of this bacterial strain (Wang et al., 2010). Additionally, several regulatory proteins involved in quorum sensing and biofilm formation, including Hfq, LuxS, and SmcR (Kim et al., 2013; Wen et al., 2016), were found to be acetylated. The role of these proteins in the regulation of virulence genes in genus *Vibrio* has also been widely reported (Waters et al., 2010; Pérez-Reytor et al., 2016), and our finding further shows the potential association between protein acetylation and the virulence of *V. vulnificus*.

### The Acetylation and Protein-Protein Interaction Network of Virulence and Antibiotic Resistance Genes

**FIGURE 6:**
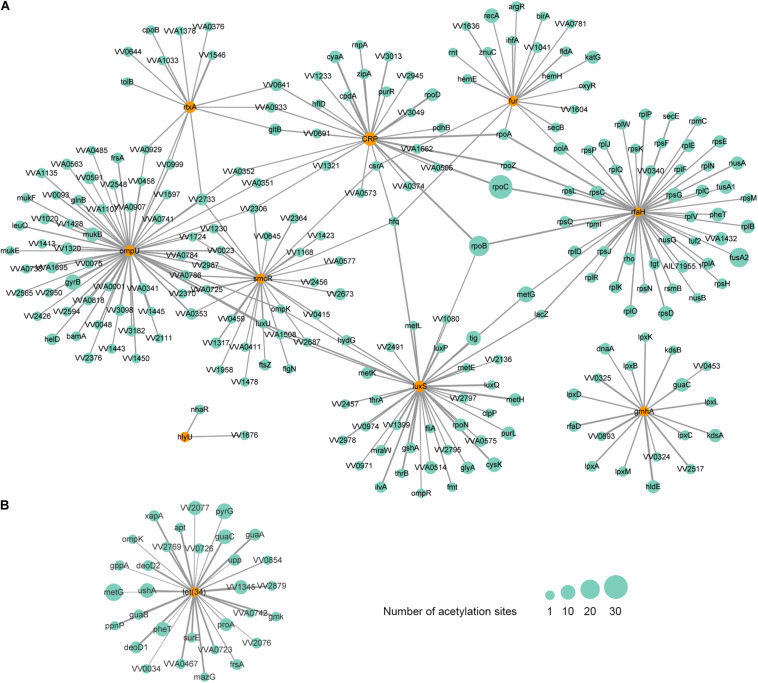
Protein-protein interaction network of acetylated virulence **(A)** and antibiotic resistance-related **(B)** factors in *V. vulnificus* Vv180806. The corresponding factors are marked in orange circles. The size of the circle represents the number of modification sites and the size of the line represents the interacted score.

As is shown above, the acetylation of LuxS and SmcR has been observed. Except for the two factors, we also found the acetylation of 7 additional VFs, including RtxA, OmpU, IlpA, Fur, CRP, HlyU, and GmhA. A protein-protein interaction (PPI) network analysis showed that these acetylated VFs are interacted with 226 additional acetylated proteins (Figure 6A). From this network, we found that RtxA directly interacts with OmpU, and both VFs are indirectly regulated by SmcR and CRP. Among them, OmpU interacts with the highest number of other acetylated proteins. In this interactive network, OmpU functions as a fibronectin-binding protein that promotes the adherence of *V. vulnificus* to the host cells (Goo et al., 2006), subsequently, the cytotoxicity conferred by RtxA will damage the intestinal mucosal barrier of the host during the bacterial contact with host cells (Liu and Crosa, 2012), and our finding suggests that lysine acetylation plays a considerable regulatory role in this process with most factors within it are acetylated. In addition, RpoC, the protein that has the highest number of acetylation sites (37 acetylation sites), is interacted with two regulatory factors (CRP and RfaH). RpoC is an RNA polymerase β′ subunit that affects RNA polymerase activity and chromosomal replication in bacteria (Petersen and Hansen, 1991; Morse et al., 1996), and it is involved in the molecular complex detection (MCODE) of ribosome according to the analysis of clustering interaction network (Supplementary Figure S7). Thus, the acetylation of RpoC might regulate the virulence of *V. vulnificus* Vv180806 by altering the expression of those upstream regulators. Overall, our findings suggest the importance of acetylation on the regulation of virulence in *V. vulnificus*, yet further studies are needed to clarify the detailed mechanism.

Interestingly, the lysine acetylation was not found in the potential cefoxitin resistance-related protein VarG, but was observed in Tet (34) that is associated with tetracycline resistance. The PPI network analysis revealed that Tet (34) interacts with 31 additional acetylated proteins (Figure 6B). The involvement of lysine acetylation in antibiotic resistance has been observed in *Salmonella* Typhimurium (Li et al., 2018). Remarkably, the lysine acetylation of some resistance-related proteins is negatively correlated with the resistance phenotypes (Li et al., 2018), indicating that the acetylation may negatively regulate antibiotic resistance genes in some cases. Thus, the fact that strain Vv180806 harbors *tet* genes but shows susceptibility to tigecycline (one of the tetracycline antibiotics) may be the result of acetylation-mediated down-regulation of *tet* gene. This deserves further study in the future.

## Conclusion

In this study, we examined the genomic features in a *V. vulnificus* strain Vv180806 that was isolated from a mortality case in China. Several factors associated with virulence and antibiotic resistance were identified in the genome sequence, and the comparative genomic analysis supported the potentially food-borne infection of this bacterial strain. After that, we investigated the global lysine acetylation in this strain. A total of 6,626 acetylation sites on 1,924 proteins were identified. These acetylated proteins target diverse functions ranging from the control of metabolic process to binding functions. Moreover, a remarkable association between acetylated modification and virulence and antibiotic resistance of this bacterium were found. Altogether, this work provides a valuable resource for diagnosing the risk of *V. vulnificus* infection in the future, and renders further investigations on mechanisms responsible for the pathogen-host interactions in this increasingly pathogen.

## Data Availability Statement

The whole genome sequence of *V. vulnificus* Vv180806 has been deposited in GenBank under accession numbers CP044206–CP044208. All raw reads of the *V. vulnificus* Vv180806 genome have been deposited in the NCBI Read Archive under accession number SRR10161471.

## Author Contributions

RP, YiL, KL, and QW conceived and designed the study. KL and PG contributed to materials. YaL, XY, SZ, TL, JW, MC, SW, and LX performed the experiments. RP and YiL performed the data analysis. RP, YiL, KL, and QW wrote the manuscript. All authors contributed to the article and approved the submitted version.

## Conflict of Interest

The authors declare that the research was conducted in the absence of any commercial or financial relationships that could be construed as a potential conflict of interest. The handling editor declared a shared department with several authors, KL and PG, at the time of the review.

## Footnotes

1 http://broadinstitute.github.io/picard/

2 https://string-db.org/cgi/input.pl?sessionId
