# Supplementary material for: Genome- and Proteome-Wide Analysis of Lysine Acetylation in Vibrio vulnificus Vv180806 Reveals Its Regulatory Roles in Virulence and Antibiotic Resistance
Source: Front Microbiol. 2020 Nov 5;11:591287. doi: 10.3389/fmicb.2020.591287 (PMC7674927; doi:10.3389/fmicb.2020.591287)
Supplement: Supplementary file 11 [file Data_Sheet_1.docx]

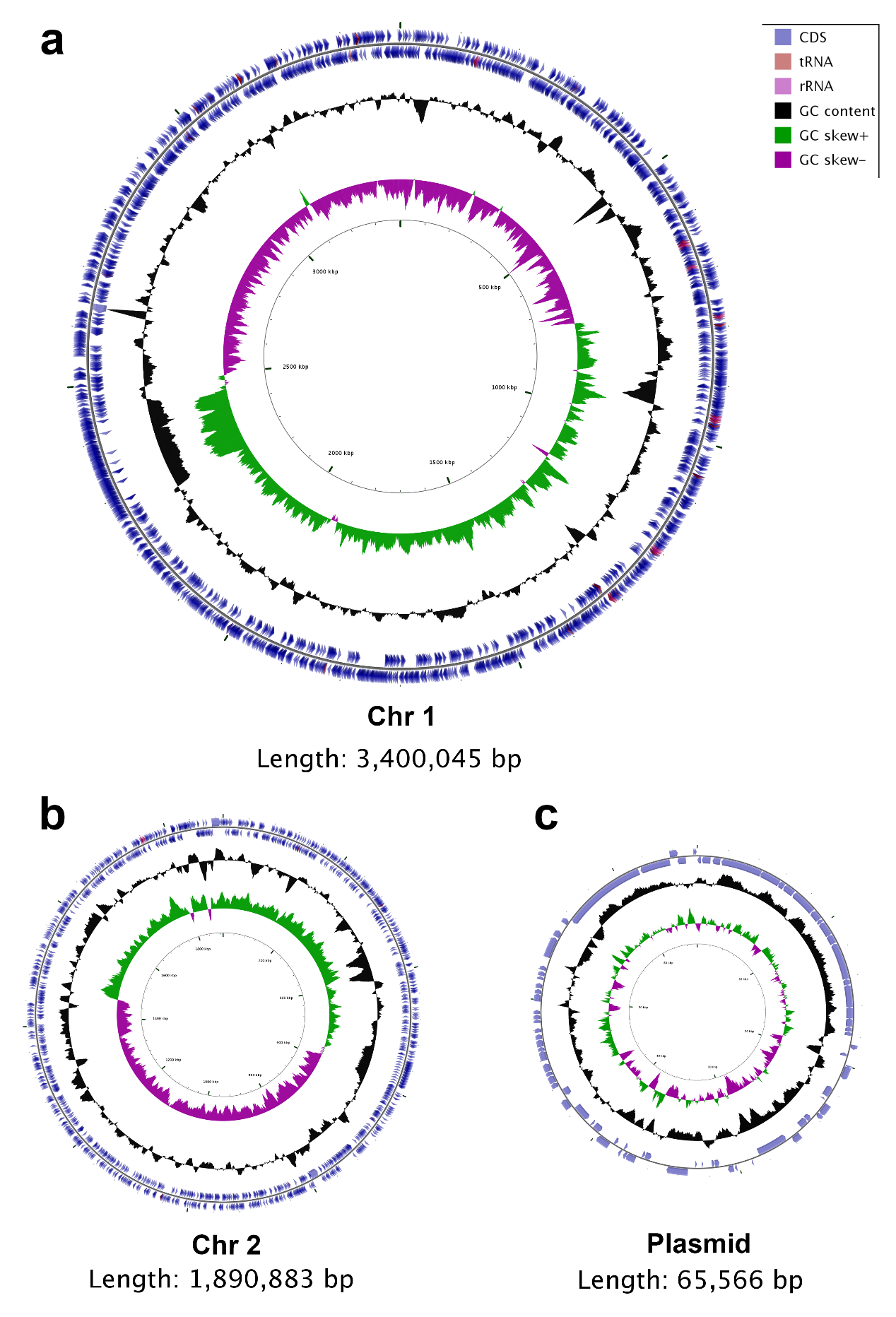


**Figure S1.** Circular representation of the genome of *V. vulnificus* Vv180806. (a) Chromosome 1; (b) Chromosome 2; (c) Plasmid. The outer two rings (ring 1 and ring 2) represent the annotated genes encoding proteins and structural RNAs on the plus and minus strands, respectively. Ring 3 (black circle) indicates the GC content (%), and ring 4 depicts the GC skew.


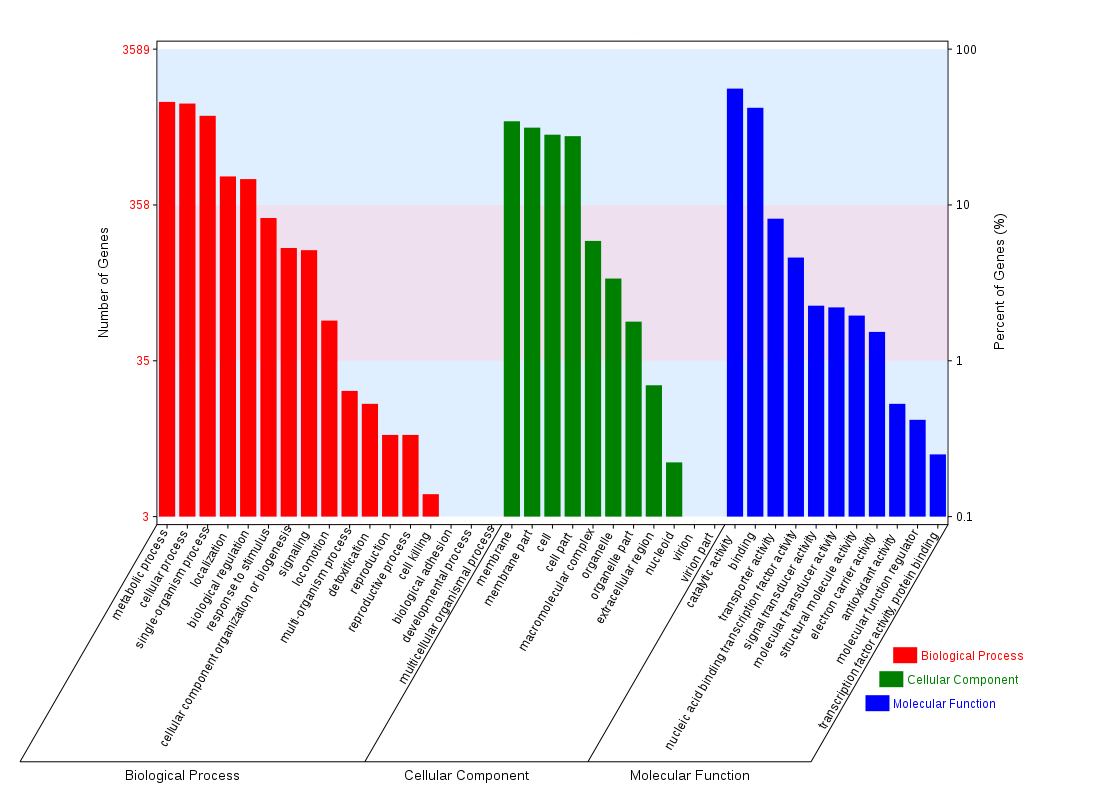


**Figure S2.** Gene Ontology (GO) functional categories of the protein coding genes.


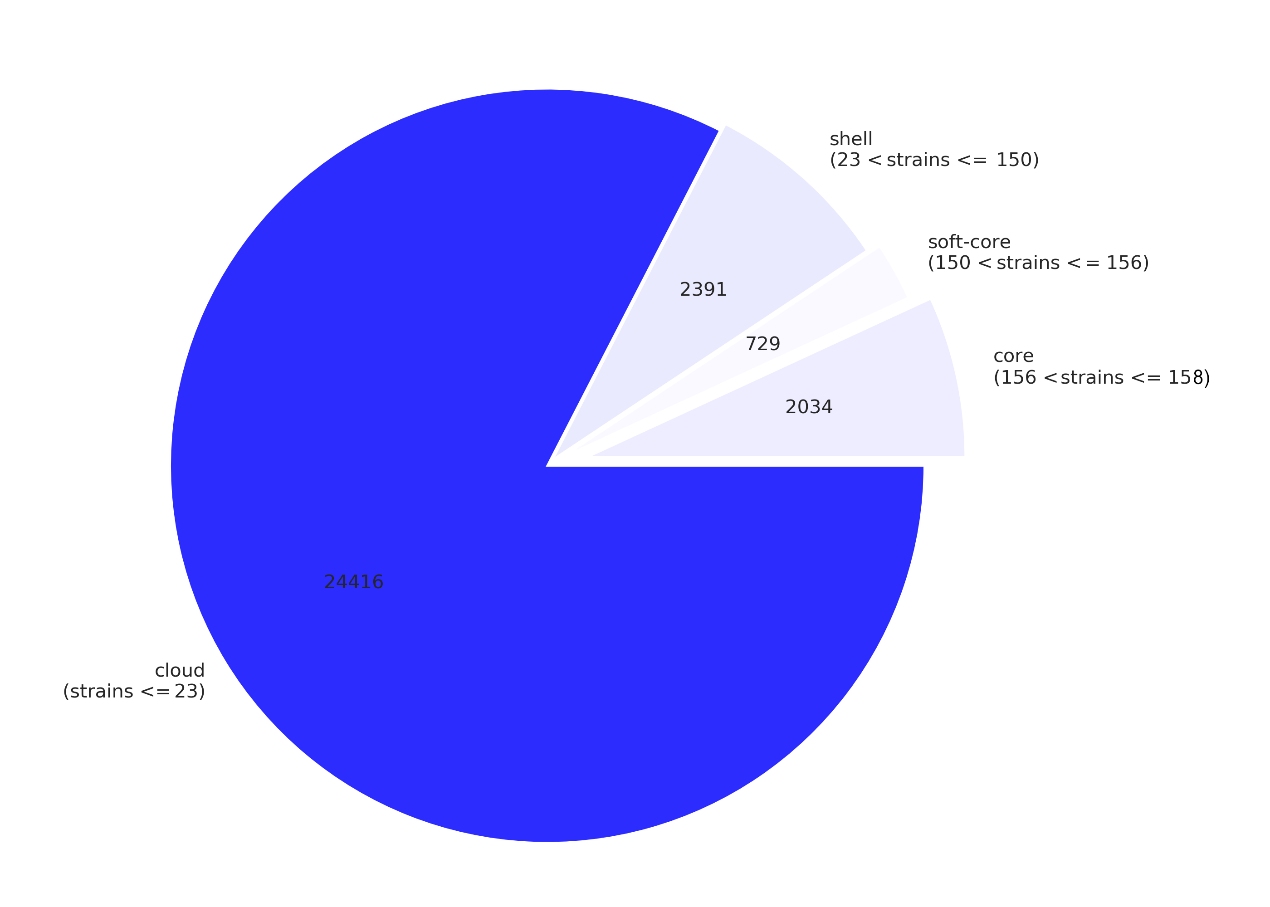


**Figure S3.** Pan-genome analysis statistics of *V. vulnificus* strains.


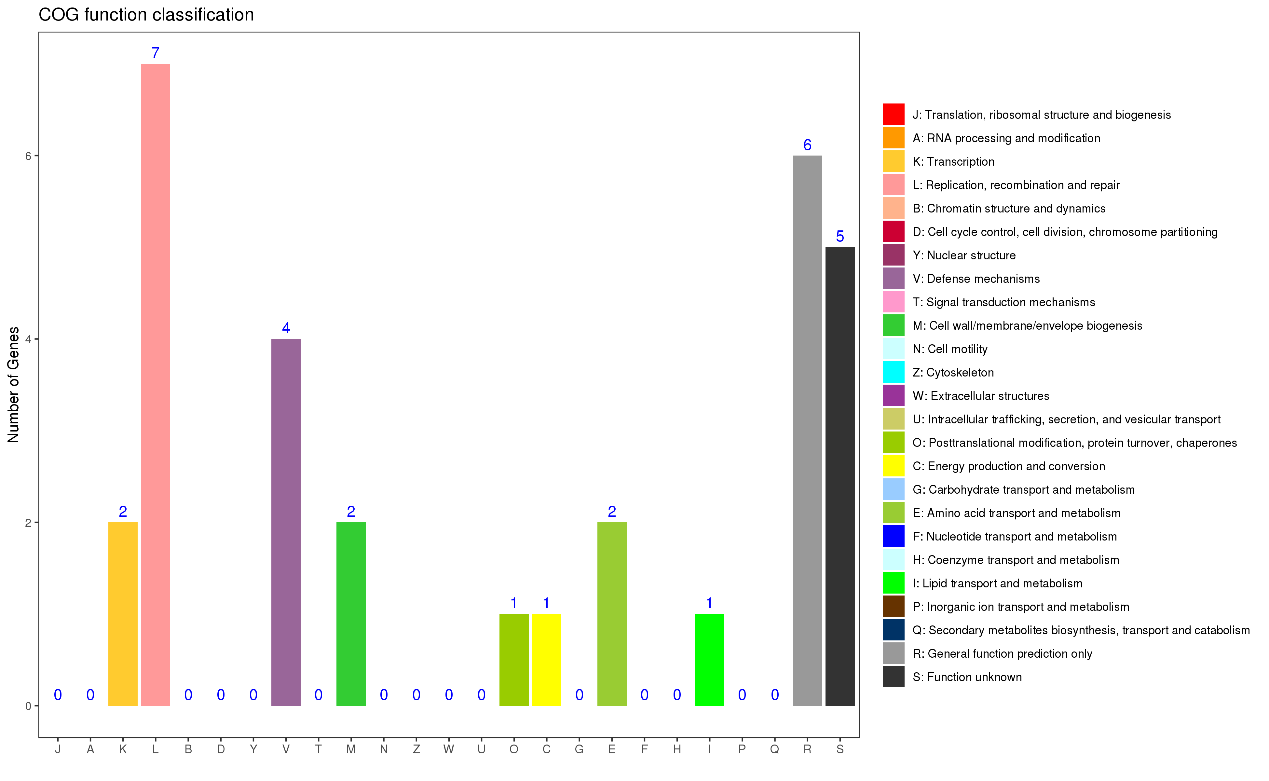


**Figure S4.** Functional classification of the strain-specific genes in *V. vulnificus* Vv180806 (COG classification).


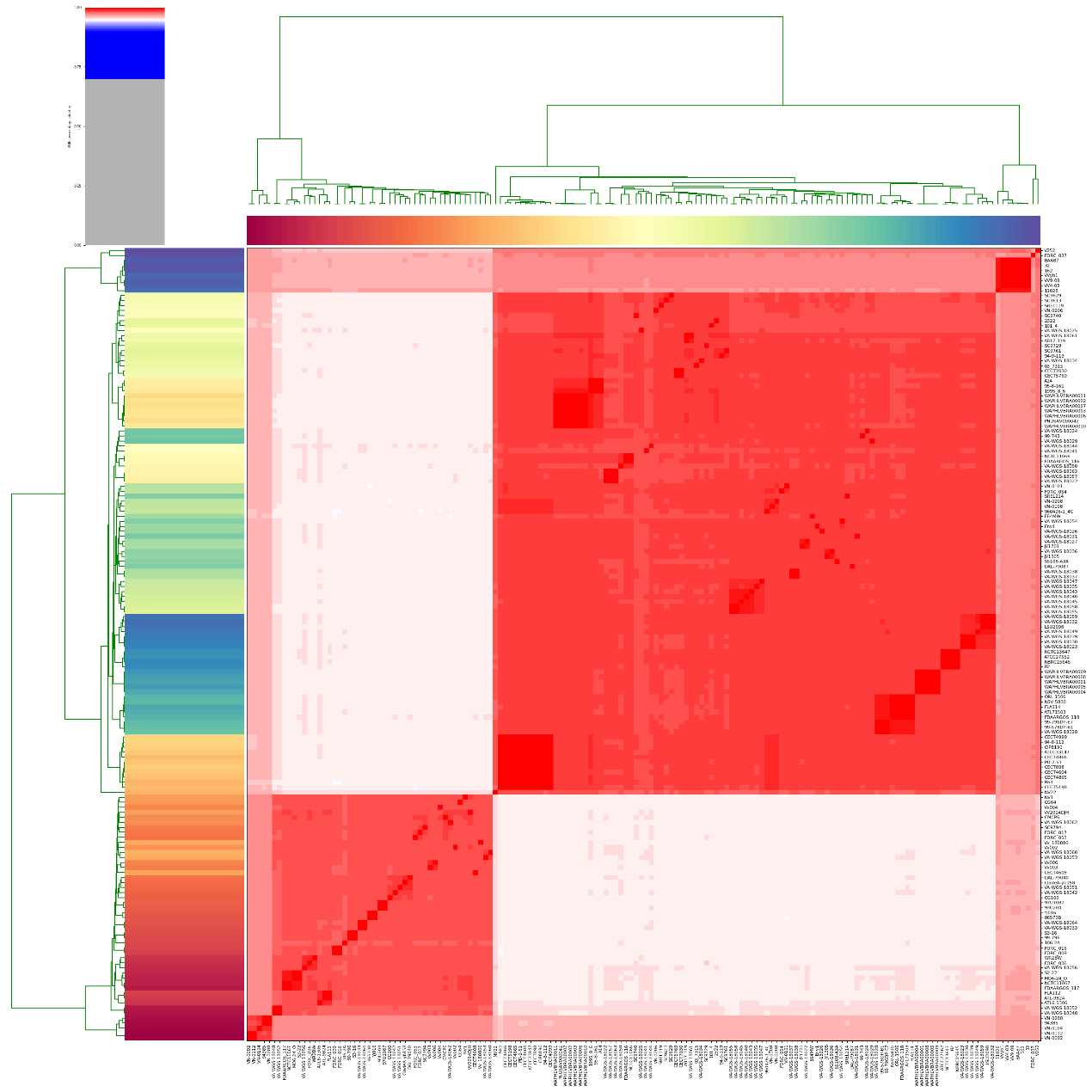


**Figure S5.** Hierarchical clustering in two dimensions of pairwise average nucleotide identity (ANI) comparison of *V. vulnificus* strains. The ANI values are presented as a heatmap generated according to the matrix of percentage identity.





**Figure S6.** Acetylation motifs and conservation of acetylation sites. The height of each letter corresponds to the frequency of that amino acid residue in that position. The central K refers to the acetylated lysine. Acetylated lysine motif are analyzed by using Motif-X software.


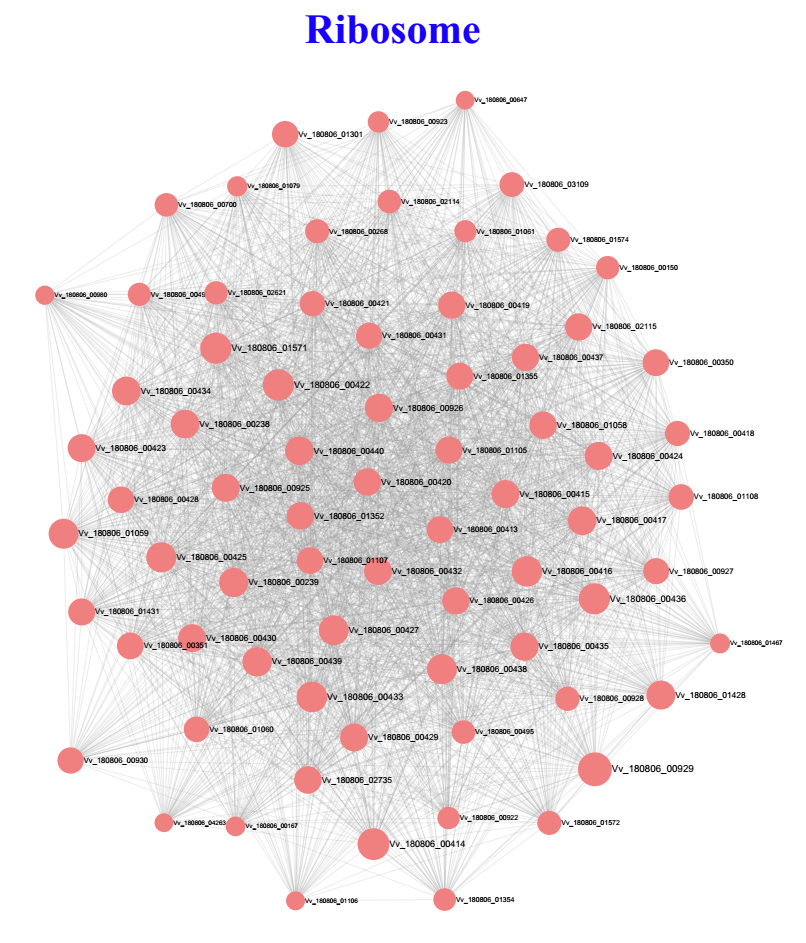


**Figure S7.** Protein-protein interaction network of acetylated proteins associated with ribosome. Gene Vv_180806_00930 encodes the RpoC.
